# CAR-T-Zell-Therapie beim multiplen Myelom

**DOI:** 10.1007/s00108-021-01043-8

**Published:** 2021-05-03

**Authors:** X. Zhou, H. Einsele, S. Danhof

**Affiliations:** Medizinische Klinik und Poliklinik II, Universitätsklinikum Würzburg, Oberdürrbacher Straße 6, 97080 Würzburg, Deutschland

**Keywords:** Adoptive Immuntherapie, T‑Lymphozyten, Chimäre Antigenrezeptoren, „B-cell maturation antigen“, Unerwünschte Wirkungen der CAR-T-Zell-Therapie, Immunotherapy, adoptive, T‑lymphocytes, Receptors, chimeric antigen, B‑cell maturation antigen, CAR T‑cell therapy/adverse events

## Abstract

Die Behandlung mit CAR-T-Zellen (*CAR* chimärer Antigenrezeptor) ist eine neuartige Strategie der zellulären Immuntherapie, die das patienteneigene Immunsystem als „Waffe gegen Tumorzellen“ benutzt. Bei Patienten mit multiplem Myelom werden CAR-T-Zell-Therapien im Rahmen klinischer Studien getestet. Die aktuellen Studiendaten der gegen das „B-cell maturation antigen“ (BCMA) gerichteten CAR-T-Zell-Therapien zeigen eine beachtliche Wirksamkeit, die eine baldige Zulassung erwarten lässt. Allerdings erleiden weiterhin die meisten Patienten nach einer Behandlung mit CAR-T-Zellen ein Rezidiv. Hinzu kommt, dass CAR-T-Zell-Therapien zu schwerwiegenden Nebenwirkungen wie Zytokinfreisetzungssyndrom und Neurotoxizität mit teilweise auch letalem Ausgang führen können. Ein angemessenes Kosten-Nutzen-Verhältnis der CAR-T-Zell-Therapie stellt eine weitere Herausforderung dar. Trotz dieser Limitationen erscheint die CAR-T-Zell-Therapie eine attraktive Option für Patienten mit Myelom, sodass diese Therapie das Potenzial hat, in die Standardbehandlung integriert zu werden.

## Abstract (en)

Chimeric antigen receptor (CAR) T-cell treatment is a novel immunotherapy utilizing the patientʼs own immune system as a “weapon against tumor cells”. In patients with multiple myeloma (MM), CAR T-cell therapy has been investigated in clinical trials. The current data on B‑cell maturation antigen (BCMA)-targeted CAR T-cells have shown impressive efficacy, and official approval is expected shortly. However, the majority of patients relapse after CAR T-cell therapy. Moreover, the treatment can cause severe adverse events such as cytokine release syndrome and neurotoxicity with lethal outcome. The cost–benefit ratio of this treatment also needs to be optimized. Despite these limitations, CAR T-cell therapy represents an attractive option for patients with MM and has the potential to be incorporated into the standard of care.

Nach über 30 Jahren präklinischer Entwicklung haben die CAR-T-Zellen (*CAR* chimärer Antigenrezeptor) mit der Zulassung der ersten CD19-gerichteten Produkte nun auch Eingang in die klinische Routine gehalten. Da der Erkrankungsverlauf beim multiplen Myelom (MM) trotz beachtlicher therapeutischer Fortschritte weiterhin zumeist von wiederholten Rezidiven geprägt ist, liegt eine Erprobung von CAR-T-Zellen zur Eliminierung maligner Plasmazellen auf der Hand.

## Status quo und therapeutische Herausforderungen beim multiplen Myelom

Das MM, eine maligne Plasmazelldyskrasie, macht etwa 10 % aller hämatologischen Neoplasien aus. Wenngleich die Erkrankung in vielen Fällen als unheilbar gilt, hat sich die Prognose für Betroffene in den letzten Jahren wesentlich verbessert. Entscheidend hierfür war der Einzug der monoklonalen Antikörper in die Standardtherapie. So erzielt die Induktionstherapie mit dem CD38-gerichteten Antikörper Daratumumab in Vierfachkombination mit Bortezomib, Dexamethason und Thalidomid bzw. Lenalidomid ein progressionsfreies Überleben („progression free survival“ [PFS]) von über 90 % nach 18 [1] bzw. 24 Monaten [2].

Dennoch bleibt bei über einem Drittel der Patienten trotz optimalen Therapieansprechens eine minimale Resterkrankung („minimal residual disease“ [MRD]) nachweisbar, sodass Rezidive erwartbar sind und Folgetherapien weiterhin benötigt werden. Insbesondere für Patienten mit Hochrisikoerkrankung – das heißt in Stadium III nach Revised International Staging System (R-ISS; [3]), mit extramedullärer Erkrankung („extramedullary disease“ [EMD]; [4]) oder ungünstigem Genexpressionsprofil [5] – sind daher neue Therapiealternativen erforderlich.

Besonders Patienten mit multiplem Myelom und Hochrisikokonstellation benötigen neue Therapiealternativen

Der erfolgreiche Einsatz von mit CAR modifizierten T‑Zellen (CAR-T-Zellen) bei B‑Zell-Neoplasien hat die Entwicklung von CAR-T-Zellen für die Behandlung anderer Krebserkrankungen derart beflügelt, dass für die Behandlung des MM gegenwärtig über 100 klinische Studien zur Untersuchung von CAR-T-Zellen registriert sind (https://www.clinicaltrials.gov).

## Zielantigene für CAR-T-Zellen beim multiplen Myelom

Das „B-cell maturation antigen“ (BCMA), auch bekannt als TNFRSF17 oder CD269, ist ein Membranprotein, das von reifen B‑Zellen und sowohl gesunden als auch malignen Plasmazellen exprimiert wird. BCMA spielt eine signifikante Rolle bei der Proliferation der malignen Plasmazellen und kann dadurch den Erkrankungsprogress fördern. Auf der Zelloberfläche normaler hämatopoetischer Stammzellen oder gesunder Gewebe ist BCMA dagegen kaum oder nicht exprimiert. Deswegen stellt es ein sehr geeignetes Zielantigen für die CAR-T-Zell-Therapie bei Patienten mit MM dar. Zurzeit ist die BCMA-gerichtete CAR-T-Zell-Therapie die am häufigsten untersuchte zelluläre Immuntherapie bei Patienten mit MM.

Prinzipiell sind andere Antigene mit Expression auf den malignen Plasmazellen ebenfalls mögliche Zielstrukturen für die CAR-T-Zell-Therapie beim MM. Präklinische und klinische Daten zeigten eine beachtliche Wirksamkeit von CAR-T-Zellen gegen CD138 (Syndecan‑1; [6]), CD19 [7], CD38 [8], κ‑Leichtketten [9], SLAMF7 (auch bekannt als CS1 oder CD319; [10]), GPRC5D [11], CD44v6 [12] und NKG2D [13]. CAR-T-Zell-Therapien gegen sämtliche der oben genannten alternativen Antigene werden derzeit im Rahmen klinischer Studien weiter evaluiert.

## Klinische Studiendaten

Die aktuell verfügbaren Daten für den Einsatz von CAR-T-Zellen zur Behandlung des MM stammen in erster Linie aus klinischen Studien, die BCMA-gerichtete CAR-T-Zell-Therapien untersuchen [14]. So sind derzeit über 10 verschiedene BCMA-CAR-T-Zell-Produkte evaluiert bzw. publiziert. Insgesamt konnten BCMA-CAR-T-Zell-Therapien eine sehr gute Wirksamkeit mit einer hohen Gesamtansprechrate („overall response rate“ [ORR]) von bis zu 100 % bei Patienten mit rezidiviertem/refraktärem MM erzielen [15]. Besonders erwähnenswert sind hierbei die Behandlungsergebnisse der KarMMa-Studie, in der Idecabtagen-Vicleucel (ide-cel, bb2121) in einem stark vorbehandelten Patientenkollektiv eine ORR von 73 % und ein mittleres PFS von 8,8 Monaten erzielte. Ebenfalls für Aufsehen sorgte die CARTITUDE-1-Studie, in der der Einsatz von LCAR-B38M im fortgeschrittenen Rezidiv zu einer ORR von 100 % und einer PFS-Rate von 86 % nach 9 Monaten führte. Die Daten einer aktualisierten Metaanalyse zeigten für 15 BCMA-CAR-T-Zell-Therapiestudien insgesamt eine hohe ORR von 82 % mit einer Rate an Komplettremissionen („complete remission“ [CR]) von 36 % [16]. Auch bei Patienten mit Hochrisikomyelom und EMD wurde eine beachtlich hohe ORR von 78 % berichtet, die mit sonstigen modernen Immunchemotherapien, beispielsweise Carfilzomib- oder Daratumumab-haltigen Regimen, nicht erreicht werden konnte [17, 18]. Diese vielversprechenden Ergebnisse aus klinischen Studien könnten zu einer baldigen Zulassung der BCMA-CAR-T-Zell-Therapie bei Patienten mit MM führen.

BCMA-CAR-T-Zell-Therapien zeigten sehr gute Wirksamkeit bei rezidiviertem/refraktärem multiplem Myelom

Im Gegensatz zur BCMA-gerichteten CAR-T-Zell-Therapie ist die Entwicklung von Nicht-BCMA-CAR-T-Zellen noch weniger fortgeschritten. In klinischen Studien wurden weniger überzeugende Wirksamkeitsdaten für CD138-, NKG2D- und κ‑Leichtketten-gerichtete CAR-T-Zell-Therapien berichtet [9, 13, 19]. In einer Studie von Garfall et al. [20] wurde eine hohe ORR von 90 % nach CD19-gerichteter CAR-T-Zell-Therapie bei Patienten mit rezidiviertem MM berichtet. Allerdings hatten diese Patienten eine zusätzliche Hochdosistherapie mit Melphalan und autologer Stammzelltransplantation vor der CAR-T-Zell-Infusion erhalten [20]. Ergebnisse einer an unserem Zentrum initiierten Phase-I-Studie mit SLAMF7-gerichteten CAR-T-Zellen werden im Jahr 2021 erwartet (NCT04499339). Die klinischen Studiendaten ausgewählter CAR-T-Zell-Therapien beim MM sind in Tab. 1 zusammengefasst.

**Table 1:** 

Zielantigen	Studie	Phase	*N*	Lymphozytendepletierende Chemotherapie	Dosis (Zellen/kg)	Anzahl der Therapielinien	Gesamtansprechrate	PFS (Median)
BCMA	NCT02215967	1	24	Cy/Flu	0,3–9 ∙ 10^6^	9,5	81 %^a^	31 Wochen
		1	12	Cy/Flu	0,3–9 ∙ 10^6^	7	100 %^a^	NA
	NCT02546167	1	25	Cy oder keine	Kohorten 1 und 3: 1–5 ∙ 10^8^ Kohorte 2: 1–5 ∙ 10^7^	7	Kohorte 1: 44 % Kohorte 2: 20 % Kohorte 3: 64 %	Kohorte 1: 65 Tage Kohorte 2: 57 Tage Kohorte 3: 125 Tage
	NCT02658929	1	33	Cy/Flu	50, 150, 450 oder 800 ∙ 10^6^	7–8	85 %	11,8 Monate
	NCT03090659	1	57	Cy	0,07–2,1 ∙ 10^6^	3	88 %	15 Monate
		1	17	Cy/Flu oder Cy	0,21–1,52 ∙ 10^6^	4	88 %	12 Monate
	NCT03430011	1/2	19	Cy/Flu	50 ∙ 10^6^ oder 150 ∙ 10^6^	10	100 %	NA
		1/2	51	Cy/Flu	300, 450 oder 600 ∙ 10^6^	6	91 %	NA
	NCT03070327	1	11	Cy/Flu oder Cy	72, 137, 475 oder 818 ∙ 10^6^	6	64 %	NA
	NCT03338972	1	7	Cy/Flu	5 oder 15 ∙ 10^7^	8	100 %	NA
	NCT03288493	1/2	25	Cy/Flu	0,5–5 ∙ 10^8^	7	48 %	NA
	NCT03274219	1	22	Cy/Flu	150, 450, 800 oder 1200 ∙ 10^6^	7	83 %	NA
	NCT03548207	1b/2	29	Cy/Flu	Median: 0,73 ∙ 10^6^	5	100 %	NA
	NCT03361748	2	128	Cy/Flu	150–450 ∙ 10^6^	6	73 %	8,6 Monate
	NCT03661554	1	16	Cy/Flu	2–10 ∙ 10^6^	NA	100 %	NA
	NCT03093168	1	46	Cy/Flu	9 ∙ 10^6^	NA	79,6 %	15 Monate
	NCT03716856 NCT03302403 NCT03380039	1	24	Cy/Flu	1,5, 0,5, 1 oder 1,8 ∙ 10^8^	4,5	87,5 %	NA
CD138	NCT01886976	1/2	5	PCD/CP/VAD	Median: 0,756 ∙ 10^7^	10	0 %	NA
CD19	NCT02135406	1	10	Mel + ASZT	1–5 ∙ 10^7^	6	90 %	200 Tage
NKG2D	NCT02203825	1	5	Keine	1 ∙ 10^6^–3 ∙ 10^7^	≥ 5	0 %	NA
κ‑Leichtketten	NCT00881920	1	7	Cy oder keine	0,92–1,9 ∙ 10^8^/m^2^	4	0 %	NA
BCMA und CD19^b^	NCT03196414	1/2	28	Cy/Flu	BCMA: 2–6,8 ∙ 10^7^ CD19: 1 ∙ 10^7^	3	92,6 %	8 Monate
BCMA und CD19^b^	NCT03455972	1/2	32	Bu/Cy oder Mel + ASZT	CD19: 1 ∙ 10^7^ BCMA: NA	NA	100 %	NA
BCMA und CD19^b^	ChiCTR-OIC-17011272	2	22	Cy/Flu	CD19: 1 ∙ 10^6^ BCMA: 1 ∙ 10^6^	6	95 %	VGPR: 243 Tage sCR: 268 Tage
BCMA/CD38^c^	ChiCTR1800018143	1	16	Cy/Flu	0,5, 1,0, 2,0, 3,0 oder 4,0 ∙ 10^6^	NA	87,5 %	NA
BCMA/CD19^c^	NA	NA	5	Cy/Flu	1,0 ∙ 10^6^ oder 2,0 ∙ 10^6^	3	100 %	NA
BCMA/TACI^c^	NCT03287804	1/2	12	Cy/Flu	15, 75, 225, 600 oder 900 ∙ 10^6^	5	43 %	NA

*ASZT* autologe Stammzelltransplantation, *BCMA* „B-cell maturation antigen“, *Bu* Busulfan, *CAR* chimärer Antigenrezeptor, *CP* Chlorambucil, Prednison, *Cy* Cyclophosphamid, *Flu* Fludarabin, *Mel* Melphalan, *NA* unbekannt, *NKG2D* „natural killer group 2 member D“, *PCD* Pomalidomid, Cyclophosphamid, Dexamethason, *PFS* „progression free survival“ (progressionsfreies Überleben), *sCR* „stringent complete remission“, *TACI* „transmembrane activator and calcium modulator and cyclophilin ligand interactor“, *VAD* Vincristin, Doxorubicin, Dexamethason, *VGPR* „very good partial remission“

^a^Bei Patienten mit der höchsten CAR-T-ZellDosis

^b^Behandelt mit BCMA- und CD19-CAR-Zellen

^c^Bi-spezifische CAR-T-Zellen

## Nebenwirkungen

Im Vergleich zur konventionellen Immunchemotherapie hat die neuartige CAR-T-Zell-Therapie ein anderes Nebenwirkungsprofil, sogar Todesfälle wurden in verschiedenen Studien berichtet. Nach der CAR-T-Zell-Infusion zeigt die Mehrzahl der Patient eine Panzytopenie, welche die häufigste hämatologische Nebenwirkung ist. Die häufigsten nichthämatologischen Nebenwirkungen bei CAR-T-Zell-Therapie sind Zytokinfreisetzungssyndrom („cytokine release syndrome“ [CRS]) und Neurotoxizität („immune effector cell-associated neurotoxicity syndrome“ [ICANS]).

Die Pathophysiologie von CRS und ICANS ist noch nicht vollständig geklärt. Ein möglicher Pathomechanismus ist eine überschießende Aktivierung der CAR-T-Zellen und anderer Immunzellen, was zu einer massiven Zytokinproduktion führen und infolgedessen Endothelschäden und Störungen der Blut-Hirn-Schranke verursachen kann. Das CRS kann sich in unterschiedlicher Ausprägung mit Fieber, Hypotonie und Hypoxie manifestieren, beim ICANS können Bewusstseinsstörung, Krampfanfälle, motorische Störungen oder sogar Hirnödeme auftreten. Auch wenn es bislang noch keine international einheitlichen Standards für die Behandlung von CRS und ICANS gibt, hat der Einsatz des Interleukin-6-Rezeptor-Blockers Tocilizumab beim CRS und von Glukokortikoiden beim ICANS einen gewissen Stellenwert erlangt.

## Resistenzmechanismen und neue Engineering-Strategien

Trotz beeindruckender Erfolge erleiden die meisten Patienten mit Myelom nach einer Behandlung mit CAR-T-Zellen ein Rezidiv. So zeigte die bereits angesprochene Metaanalyse von 15 klinischen Studien mit Patienten, die im Rezidiv mit BCMA-CAR-T-Zellen behandelt wurden [16], zwar eine beeindruckende ORR (82 %) und CR-Rate (36 %), gleichzeitig wurden aber eine Rückfallrate von 45 % und ein mittleres PFS von nur 10 Monaten dokumentiert. Ein derartiges Therapieversagen kann durch T‑Zellen oder Tumorzellen vermittelt sein. Während dem T‑Zell-vermittelten Therapieversagen häufig eine unzureichende Proliferation oder Persistenz der CAR-T-Zellen zugrunde liegt, steht beim tumorzellvermittelten Therapieversagen häufig der Verlust des Zielantigens im Vordergrund.

**Figure 1:**
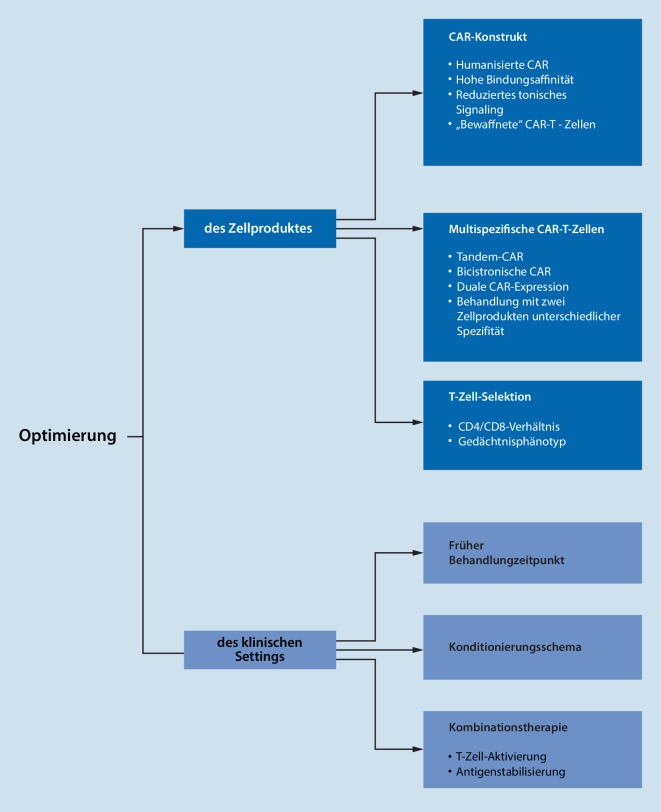

Strategien, um die Effizienz der CAR-T-Zellen weiter zu steigern, sind vielfältig (Abb. 1). Die präklinische Entwicklung fokussiert dabei auf die Konstruktion (1) voll humanisierter CAR zur Reduktion der Gefahr einer Abstoßungsreaktion gegen die CAR-T-Zellen (z. B. MCARH171 [11]) mit (2) optimierter Bindungsaffinität an das Tumorantigen (z. B. LCAR-B38M [21]) und (3) reduzierter tonischer Signalübertragung (z. B. auf Centyrin™ [Aro Biotherapeutics, Philadelphia, PA, USA] basierende CAR-T-Zell-Produkte [22]), da diese mit vorzeitiger Erschöpfung der CAR-Effektorfunktionen einhergeht. Darüber hinaus setzt sich zunehmend die Überzeugung durch, dass gezielt selektierte T‑Zell-Subpopulationen im Hinblick auf CD4/CD8-Verhältnis und Gedächtnisphänotyp einen Einfluss auf den Erfolg der CAR-T-Zell-Therapie haben [23].

Multispezifische CAR-T-Zell-Produkte sollen einem Antigenverlust auf den Tumorzellen entgegenwirken

Um der Gefahr eines Antigenverlusts auf den Tumorzellen entgegenzuwirken, werden sogenannte multispezifische CAR-T-Zell-Produkte mit Reaktivität gegen mehrere Antigene entwickelt. Ein Beispiel hierfür ist ein CAR, dessen Bindedomäne an den körpereigenen Rezeptor APRIL („a proliferation-inducing ligand“) angelehnt ist, der nicht nur BCMA, sondern gleichzeitig ein weiteres Tumorantigen namens TACI („transmembrane activator and calcium modulator and cyclophilin ligand interactor“) bindet [24] und somit die Wahrscheinlichkeit des Verlusts der Zielantigene reduziert. Andere bispezifische CAR-T-Zell-Produkte erkennen neben BCMA die Antigene CD19, CD38 oder SLAMF7 und befinden sich gegenwärtig in klinischer Testung. Vorläufige Daten aus einer Phase-1-Studie von BCMA/CD38-bispezifischen CAR-T-Zellen zeigen eine sehr hohe ORR von 87,5 %, und alle Patienten mit EMD haben eine Remission (partielle Remission oder besser) erreicht [25].

Neben Strategien, die in erster Linie auf eine optimierte Konstruktion der CAR-T-Zellen abzielen, gewinnt die Exploration des idealen klinischen Settings für die Behandlung zunehmend an Bedeutung. Durch oft langjährige intensive chemotherapeutische Vorbehandlung scheint die Fitness der T‑Zellen von Patienten mit (mehrfach) rezidivierter Erkrankung deutlich geringer zu sein als zu Erkrankungsbeginn. Um dieser Hypothese auf den Grund zu gehen, schließen klinische Studien zunehmend auch nicht (z. B. KarMMa-4-Studie) oder nur gering vorbehandelte Patienten (z. B. CARTITUDE-2-Studie) ein. Auch die Frage nach geeigneten Kombinationstherapeutika wird vermehrt adressiert, insbesondere mit Blick auf Medikamente, welche die Antigenexpression auf Tumorzellen stabilisieren (etwa γ‑Sekretase-Inhibitoren, NCT03502577) oder gleichermaßen myelomdepletierende und T‑Zell-aktivierende Effekte aufweisen (z. B. Lenalidomid, NCT03070327).

## Praktische Therapiedurchführung und Therapiekosten

Im Unterschied zu den B‑Zell-Neoplasien hat bislang kein CAR-T-Zell-Produkt für die Behandlung des MM eine Zulassung durch die US Food and Drug Administration (FDA) oder Europäische Arzneimittel-Agentur (EMA) erhalten. Der Einsatz erfolgt daher bis auf Weiteres nur im Rahmen klinischer Studien. Diese Studien werden den Nachweis erbringen müssen, dass die einmalige Behandlung mit CAR-T-Zellen der kontinuierlichen Standardmyelomtherapie überlegen ist, insbesondere im langfristigen Verlauf. In Anbetracht der enormen Fortschritte der letzten Jahre in der konventionellen Myelomtherapie hängt damit die Messlatte für eine Zulassung der CAR-T-Zellen beim MM ausgesprochen hoch. Zudem ist für einen breiten Einsatz ein angemessenes Kosten-Nutzen-Verhältnis erforderlich, wobei die Prognosen einen ähnlichen Kostenrahmen wie bei den CD19-CAR-T-Zell-Produkten vorsehen (300.000–500.000 $). Sollten die CAR-T-Zellen einen relevanten Anteil an Patienten vom MM heilen können, dürfte dies allerdings kein Hindernis darstellen.

## Fazit für die Praxis

- Die CAR-T-Zell-Therapie (*CAR* chimärer Antigenrezeptor) ist eine hochwirksame Behandlung für Patienten mit multiplem Myelom. Die baldige Zulassung eines ersten gegen das „B-cell maturation antigen“ (BCMA) gerichteten Zellprodukts wird erwartet.
- Aktuell sind in Deutschland CAR-T-Zell-Behandlungen von Patienten mit Myelom nur im Rahmen entsprechender Studien an geeigneten Zentren möglich.
- Die Verlängerung der Remissionsdauer ist aktuell die größte Herausforderung in der Entwicklung der CAR-T-Zell-Therapie beim MM.
- Für eine breite Anwendung ist ein akzeptables Kosten-Nutzen-Verhältnis der CAR-T-Zell-Therapie Voraussetzung.
